# Biofilm Formation and Structure in the Filamentous Fungus Fusarium graminearum, a Plant Pathogen

**DOI:** 10.1128/spectrum.00171-22

**Published:** 2022-08-11

**Authors:** Rebecca Shay, Aaron A. Wiegand, Frances Trail

**Affiliations:** a Department of Plant Biology, Michigan State University, East Lansing, Michigan, USA; b High School Honors Science-Engineering-Mathematics Research Program, Michigan State University, East Lansing, Michigan, USA; c Department of Plant, Soil and Microbial Sciences, Michigan State University, East Lansing, Michigan, USA; Beijing Forestry University

**Keywords:** *Fusarium*, biofilms, mycology, plant pathogens

## Abstract

Biofilms are protective structures for pathogens of plants and animals, in which cells are shielded from host defense responses and antimicrobial treatments. Although biofilms are well studied in bacterial pathogens, their development and structure in filamentous fungi, as well as their role in pathogenicity, are poorly understood. We show that the economically important plant pathogen Fusarium graminearum, a filamentous fungus, forms biofilms *in vitro,* which adhere to polystyrene, a hydrophobic surface. The biofilms have complex hyphal structures surrounded by a polymeric matrix that consists primarily of polysaccharides and extracellular nucleic acids, and lack lipids. Pellicles are formed in liquid cultures, floating biofilm masses that are common in bacterial biofilms, and noted but undescribed in filamentous fungal biofilms. Commonly, F. graminearum grows as hyphal colonies; however, on media which lack electron acceptors, an altered morphology is formed with predominantly short, bulbous hyphae embedded in the matrix. Supplementation of the biofilm-inducing medium with an electron acceptor restores the filamentous hyphal morphology, demonstrating that the formation of bulbous hyphae is due, at least in part, to oxidative stress. Plant hosts infected with pathogens generally respond by producing reactive oxygen species, commonly produced as a defense response. Thus, the formation of biofilms strongly suggests a role in protecting cells from host responses during the course of plant disease.

**IMPORTANCE** Fusarium graminearum is a filamentous fungal pathogen that causes Fusarium head blight (FHB) in cereal crops, leading to devastating crop losses. We have demonstrated the ability of this pathogen to form biofilms. Biofilms are likely to be important in the disease cycle of F. graminearum and other plant pathogens, protecting cells from plant defenses and environmental conditions. Towards this end, we have characterized the formation of biofilms in F. graminearum *in vitro*, which, together with ongoing characterization of their association with host plants, provides a basis for understanding the functionality of biofilms in the pathogen disease cycle.

## INTRODUCTION

Biofilms are three-dimensional structures formed by microbes, composed of complexes of living and dead cells embedded in an extracellular matrix and adhered to their surroundings (1). Biofilms formed by bacterial and yeast pathogens cause many health problems in human and plant systems, and profoundly affect our ability to control disease. Instead of surviving as individual free-living cells, microbes form organized collaborative communities to increase survival in a changing environment, including shifting salinity, pH, desiccation, physical instability, and chemistry (2). For example, xylem-dwelling microbes withstand the flow of xylem sap by adhering to surfaces to avoid being washed out of the system (3). Yeasts adhere to medical tubing as biofilms (4–7). Indeed, studies of bacterial biofilms have shown that compared to free-living cells, bacterial biofilms are 10- to 1,000-fold less susceptible to antimicrobial agents (8). Similarly, biofilms have been hypothesized to contribute to fungicide tolerance, a problem that can be compounded as fungicides are usually tested on free-living cells, without taking into account the added resistance of biofilm communities (9).

Biofilm development in yeasts and bacteria is depicted in a four-stage model: (1) single free-living cells attach to a surface; (2) the attached cells recruit additional single cells from the surrounding area; (3) colonies form a matrix composed of extracellular polymeric substance (EPS) and internal structures differentiate; (4) cells from the mature biofilm detach and disperse (2, 7, 10, 11). This model, first developed for bacterial biofilms, has been applied to single-celled fungi such as Candida albicans as well (1, 7, 12). Biofilms have high water content in their EPS matrices, which protect cells from desiccation in surface-dwelling biofilms (13). In yeasts and bacteria, the EPS matrix consists of extracellular nucleic acids, polysaccharides, proteins, and lipids, and, along with the layered biofilm structure, protects internal cells from chemicals and other harsh conditions (1, 2, 5, 8, 11, 14–25).

Despite numerous studies of biofilms in single-celled fungi, formation by filamentous fungi is comparatively understudied (26). Biofilms of filamentous fungi have been reported in Aspergillus *sp*, Fusarium *sp*, *Botrytis sp*, and *Verticillium sp*, as well as a few plant pathogenic oomycetes (11, 15, 17, 20, 27–30). These publications describe adherence of filamentous fungi to surfaces, but do not provide the details of the structure and development of biofilms, with the exception of a report in Fusarium solani isolated from keratitis. Córdova-Alcántara et al. (2019) tracked the development of biofilms in Fusarium solani in culture, including components of the surrounding matrix. However, prior to work reported here, similar detail had not been reported in plant pathogenic fungi. To explore the role of biofilm formation in filamentous fungal plant pathogens, we studied the process in Fusarium graminearum, an economically important plant pathogen worldwide, impacting the quantity and quality of harvested grains as a causal agent of Fusarium Head Blight (FHB) (31–33). Here we report our findings on the development of biofilms *in vitro*.

## RESULTS

### Development of F. graminearum biofilms.

**FIG 1:**
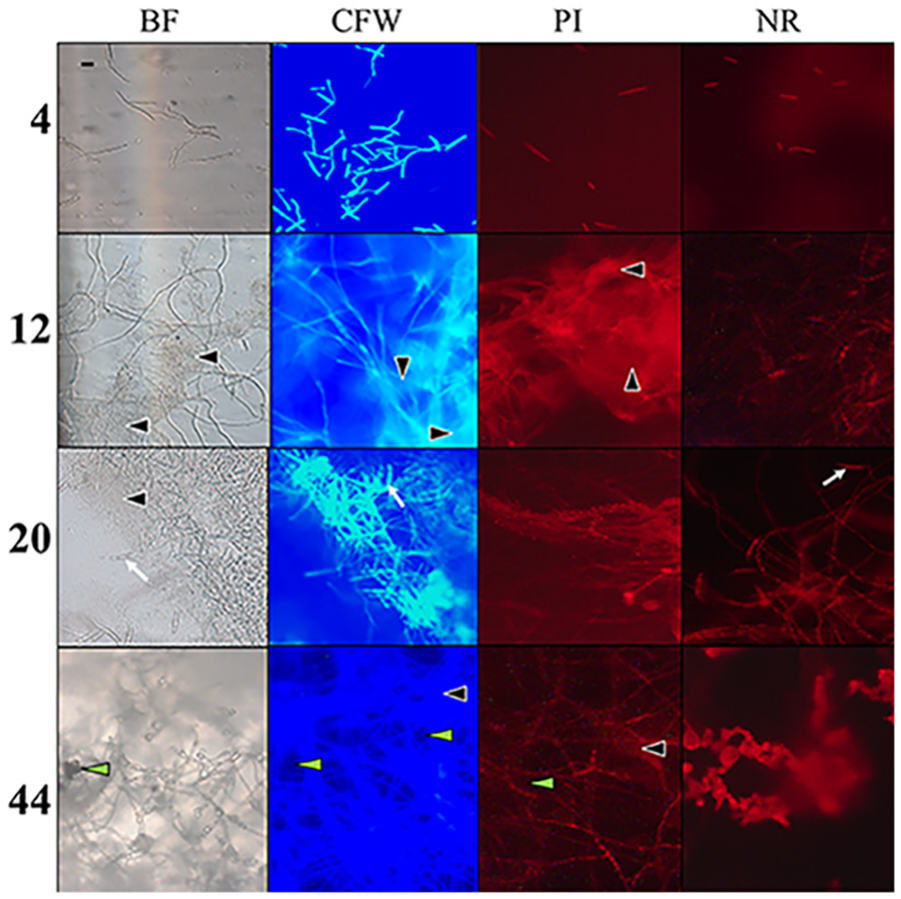
Time-course (hours) of biofilm development with identification of EPS components. Images recorded in brightfield (BF); calcofluor white (CFW), which stains polysaccharides in the cell walls and matrix; propidium iodide (PI), which stains nucleic acids; and nile red (NR), which stains lipids. Images recorded at 200× total magnification and 4 sec exposure for consistency at later time points, despite saturation. EPS matrix (black, filled arrowheads); and developing conidia (white arrows); indicate location of water channels (green arrowheads) penetrating the layers. Scale bar = 20 μm is representative of all micrographs.

**FIG 2:**
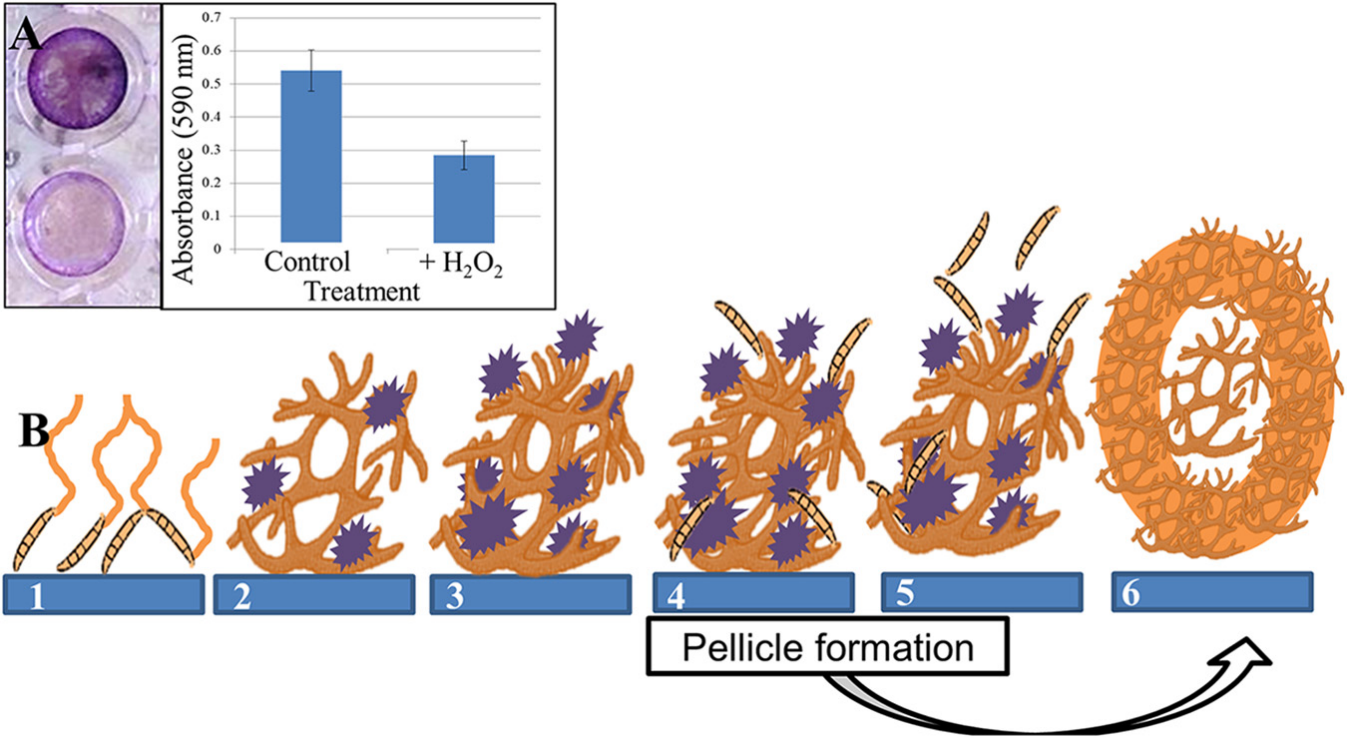
Biofilm development in Fusarium graminearum. (A) Adhesion assay for biofilm development. (Left) Cells in culture adhering to polystyrene are quantified with crystal violet stain following treatment with ROS stressor H_2_O_2_: without (top), with (bottom). (Right) Absorbance at 24 h postinoculation. (B) Model of developmental process: (1) Biofilms are initiated with the adherence of germinating conidia (orange) to the surface (blue); (2) As hyphae grow, EPS (purple) appears in clumps surrounding hyphae, contributing to complex three-dimensional structures. (3) Gaps are established through the layers of the structure for water and nutrient flow. (4) Conidial production is initiated, in preparation for dispersal. (5) At maturity, the mycelial mass no longer adheres to the surface, and can be easily removed by washing. (6) Pellicle formation in mature biofilms—floating cell clusters form thickened walls of hyphae surrounding loosely packed cells (see Fig. 5).

To document the development of biofilms in *F graminearum*, we performed adhesion assays to characterize adherence of hyphae to a polystyrene surface and the structures that form subsequently. Adhesion of germinating conidia was observed at 4 h postinoculation (hpi), and the structures continued to adhere for 44 hpi, before detaching *en masse* at 48 hpi (Fig. 1). Three dimensional structures were initiated at 12 hpi, when the EPS matrix began to develop in clumps dispersed on the hyphae, and continued to expand and surround the cell structures. Staining of the biofilm with calcofluor white exhibited the increased deposition of polysaccharides over time in the EPS matrix, and these deposits became the major component of the final EPS matrix (Fig. 1, column 2). Propidium iodide staining suggested the deposition of large quantities of extracellular nucleic acids in EPS during the early stages of biofilm formation and did not increase greatly over time (Fig. 1, column 3). In contrast, lipids were mainly localized in hyphal cells, not in the EPS matrix, and their distribution in cells increased over time (Fig. 1, column 4). Proteins, stained with Rhodamine B, appeared consistently, but in small amounts, over the developmental time course (data not shown). EPS matrix components continued to be produced as the biofilm expanded. At 20 hpi, conidia proliferated, initiating the biofilm dispersal stage. After the biofilm was fully developed (44 hpi), the large mycelial mass was released from the polystyrene surface and appeared as a floating mycelial mat. The final mass was hydrophobic on the upper surface, and hydrophilic on the lower surface that was adhered to polystyrene. When the cultures were amended with the reactive oxygen species (ROS) H_2_O_2_, the formations adhering to the surface at 24 hpi decreased significantly (*P* < 0.05) compared to cultures grown in unamended medium (Fig. 2A).

### Bulbous hyphae development.

**FIG 3:**
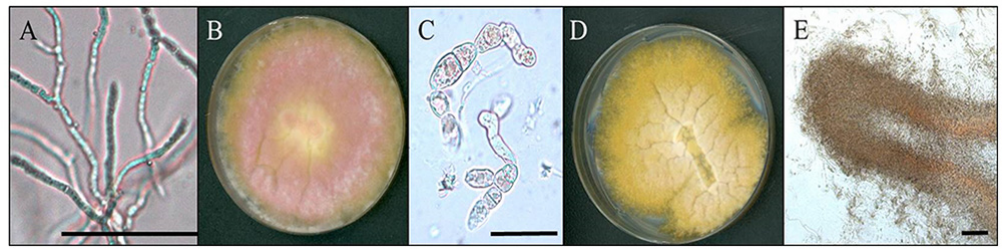
Morphology of biofilm formation under oxidative stress. (A) Normal filamentous hyphae (bar = 50 μm). (B) Growth on Bird medium agar supplemented with electron acceptor (KNO_3_) to reduce oxidative stress. (C) Bulbous hyphae produced under oxidative stress (bar = 50 μm). (D) Growth on unsupplemented Bird medium agar. Note folds in growing colony and lack of aerial hyphae (pink) compared to (A). (E) Cross section of fold in biofilm (bar = 100 μm) cut from a plate with bulbous hyphae.

**FIG 4:**
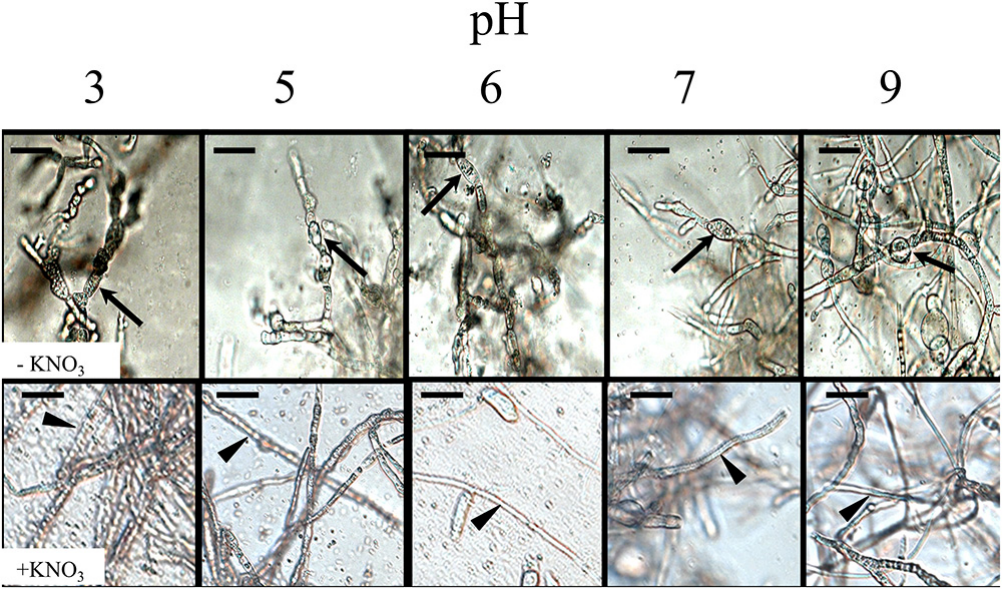
Impact of pH and electron acceptor on hyphal morphology. pH of medium (originally pH 6) was adjusted as indicated. Unamended medium (upper row). Medium amended with 20 mM KNO_3_, an electron acceptor (bottom row). Arrows indicate bulbous hyphae. Arrowheads indicate hyphae with normal morphology. Scale bar = 20 μm.

In the presence of oxidative stress, hyphae switched morphology from normal filamentous growth (Fig. 3A) to atypical bulbous cells (Fig. 3C). Wild type (WT) cells displayed bulbous hyphae when grown on solid medium or in liquid Bird medium amended with H_2_O_2_. Furthermore, when the medium was supplemented with electron acceptors, the straight hyphal phenotype was restored. In solid medium, when supplemented with an electron acceptor, the culture was fluffy, with numerous aerial hyphae (Fig. 3B). Under oxidative stress, the colony appeared wrinkled (Fig. 3D), thus increasing the overall surface area of the culture compared to WT and those supplemented with electron acceptors. A cross-section of the colony showed that the wrinkles were throughout the culture, increasing surface area in all the growth, and not just on the exposed surface of the plate (Fig. 3E). To confirm this response is due to oxidative stress and not a response to the variation in pH or nitrogen availability, the hyphal morphology was monitored on solid medium under pH ranging from 3 to 9. The bulbous hyphae were observed in all treatments at 7 dpi regardless of the pH (Fig. 4). However, when agar was supplemented with an alternative electron acceptor (either potassium nitrate or Prussian blue), normal filamentous hyphal growth was restored (Fig. 3 and 4).

### Initiation of biofilms under different conditions.

**FIG 5:**
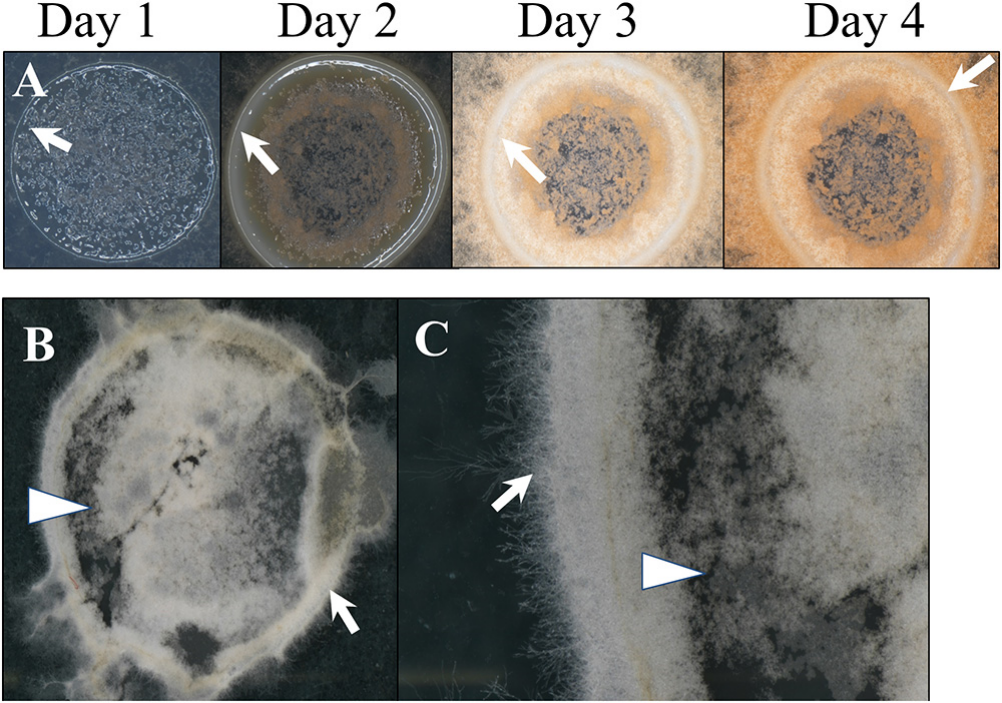
Growth at the air/liquid interface. (A) Pellicle development following inoculation with liquid conidial suspension on agar, 1 to 4 dpi (×20 magnification). (B and C) Pellicle from the top of the adhesion assay at 24 hpi (B, ×10 magnification; C, ×40 magnification). Imaging was performed on a black background, which is visible through the center of pellicles. Arrows indicate thickened ridges of cells. Arrowheads indicate regions of loosely packed cells.

Biofilm formation was observed in association with the inoculation droplet of conidia on a Bird medium agar plate. We documented two different conditions in which biofilms of filamentous hyphae were formed at the air/liquid interface. Over 4 days, a thickened ridge of cells formed at the edge of the droplet, with looser packed cells in the center of the droplet, and directly surrounding the edge of the droplet (Fig. 5A). Similar morphology was observed in biofilms grown in liquid medium in an adhesion assay. Floating pellicles of cells that adhered to each other formed at the surface of adhesion assay wells. These pellicles can be removed intact from the wells. Microscopic observations showed that pellicles have a thickened outer wall composed of individual cells and hyphae, surrounding looser cells in the middle, and a lawn of cells in the very center (Fig. 5B and C). The upper surface of these formations is hydrophobic. In both air and liquid interface environments, cellular growth shares the same morphological characteristics of densely packed cells forming a wall that surrounds looser cells to still allow nutrient flow.

## DISCUSSION

We have identified biofilm formation and investigated the process *in vitro* for the first time in F. graminearum. The process initiates in liquid culture with spore attachment to a polystyrene surface, followed by cell proliferation and formation of EPS matrix surrounding the hyphae, with subsequent copious production and dispersal of conidia, and finalizes with detachment of the biofilm from the surface. Mature biofilms lose attachment to the polystyrene surface within 48 h of initiation, suggesting that these formations may be highly transient in nature. We observed cell flocculation, resulting in pellicles, structures analogous to the early growth we observed on agar medium. We present a model for the entire developmental process of biofilms in F. graminearum (Fig. 2B) based on our *in vitro* assays, that goes beyond the commonly-used four step model (attachment, recruitment, maturation, and dispersal) of biofilm development from singled celled organisms (2, 7, 10, 11). Our model incorporates the steps from previous models along with pellicle formation, which has not been previously described in fungal biofilms.

The EPS matrix of the F. graminearum biofilm is composed of multiple macromolecules, including nucleic acids, polysaccharides, and proteins, which is consistent with the components of previously described biofilms of filamentous fungi (11, 21). The matrix components are not produced simultaneously during biofilm development, but rather are sequentially produced, suggesting that the macromolecule classes may have differential functions within the matrix. Sequential development of the matrix macromolecules has been previously demonstrated in bacterial biofilms (21), although to our knowledge, has not been observed in fungal species before this work. Nucleic acids have been identified in Pseudomonas aeruginosa and Aspergillus fumigatus, where they function as a structural component of the matrix (21, 34). In F. graminearum, extracellular nucleic acids are incorporated early in EPS matrix formation and, similarly, likely function as a scaffold, directing the complete matrix structure of the biofilm. Polysaccharides develop in large quantities as the matrix forms in F. graminearum, replacing nucleic acids as the most abundant component in the mature biofilm matrix. In fungal biofilms, polysaccharides provide protection against desiccation and nutrient loss as the biofilm develops (21). Lipids are not major components of the EPS matrix in F. graminearum, despite being an important component of C. albicans EPS matrices, rather they accumulate within the hyphae as biofilm develops. In C. albicans biofilms, lipid metabolism increases after cell adhesion as an integral matrix component (35), so the lack of lipids in the F. graminearum biofilm matrix was surprising. Understanding the role of each component of the EPS matrix will help determine how to arrest the formation of EPS components, affecting biofilm formation in filamentous fungal pathogens.

Pellicles are floating masses of cells that adhere to each other and that can be moved as a whole unit. We have shown that pellicles will form during the adhesion assay. Pellicles have been described in bacterial biofilms and in biofilms not associated with solid surfaces, where they typically occur floating at an air/liquid interface, surrounded by a matrix (36, 37). Kombucha cultures form “scobies,” consisting of fungi and bacteria adhered together in a stable form (38–40), and are transferred to initiate new fermentations. Aspergillus flavus was subcultured from a fungal pellicle that developed during a dilution of a liquid culture (41), although there was no formal description of the pellicle formation or appearance. In our study, pellicles, along with the final mature biofilm mass, have distinct surfaces—hydrophobic upper surfaces, and hydrophilic undersides. This differentiation across surfaces could assist in dispersal, where the masses potentially orient themselves in water runoff moving across aerial plant parts and soil. These formations are also lipid-rich, and the accumulated lipids in biofilms likely energetically support subsequent establishment in a new site, supporting growth and sporulation. Lipids have previously been shown to accumulate in large amounts in overwintering hyphae in crop residues, where they support the development of perithecia in F. graminearum (42).

Bacteria are induced to produce biofilms by environmental ROS to help protect cells against oxidative stress (43). In C. albicans, ROS is closely linked with quorum sensing. Quorum sensing molecules are produced in response to oxidative stress, which leads to decreased biofilm growth and potential programmed cell death (43). ROS are often produced by plants during pathogen infection, and therefore addition of ROS to cultures of F. graminearum mimics an aspect of the environment during plant infection (44). In cultures supplemented with hydrogen peroxide, we observed the formation of bulbous hyphae—a distinct morphological change in response to ROS. To confirm the cause and effect, we also assayed other possible triggers of bulbous hyphae. Nitrogen availability is dependent on pH (45); however, pH did not impact formation of bulbous hyphae, as cultures in media with a pH between 3 and 9 produced bulbous hyphae in the presence of ROS. In the presence of electron acceptors, either potassium nitrate or Prussian blue, normal filamentous hyphal growth was restored, which supports the conclusion that this phenotype is a response to oxidative stress.

The wrinkled surfaces of F. graminearum biofilm-producing cultures increase fungal surface area, and are analogous to structures characteristic of bacterial biofilms. Wrinkled surfaces with increased folds and crevices facilitate the absorption of nutrients, especially from fluids in which cultures may be growing (36, 46). Cross sections of F. graminearum cultures revealed that the colonies were composed of bulbous cells, a distinct and unusual phenotype. Increased surface area of the colony allows cells to produce more adhesion proteins for better adherence to surfaces, and increases the ability to uptake substances from the environment to combat external stressors and increase nutrient utilization. Surprisingly, the adhesion potential of the WT decreases when the culture is amended with H_2_O_2_ at low concentration. It is likely that while ROS play a role in biofilm formation, it may not be the signal initiating formation, but instead the concentration of ROS could be the signal to induce senesce of the biofilms and release cells to disperse from a toxic environment. Initial adhesion may be involved in plant infection, and a ROS burst from the plant may trigger the disassembly of biofilm formation to continue the infection processes. The complex relationship of ROS and biofilms is not entirely understood and we are only beginning to elucidate this process in F. graminearum.

The temporary nature of biofilm formations adhering to a surface indicates that there are signals to dissolve the formations and continue the life cycle elsewhere. Using *in vitro* studies of F. graminearum biofilm formation will form the basis for future biofilm formation studies *in planta*. Generally, stable biofilms will increase the pathogen’s tolerance of plant defense responses such as ROS. Even if some cells from the structure die, the dead cells and the outside matrix can act as a physical shield to protect the other cells. Increased tolerance of biofilms to plant defenses and fungicides needs to be considered in both medical and agricultural settings for effective disease control. Biofilms may play a strong ecological role in Fusarium Head Blight disease development, and new targets can be identified to improve disease control of biofilm-forming filamentous fungal pathogens.

## MATERIALS AND METHODS

### Strains and culture conditions.

F. graminearum wild-type (WT) strain PH-1 (FGSC 9075) (47) was stored short-term as conidial stocks (1× 10^7^ conidia/mL) and maintained in long-term storage as colonized agar blocks in 35% glycerol at −80^o^C. Conidia were generated in liquid carboxymethyl cellulose medium (48) with incubation at room temperature (RT) (22–25^o^C), shaking at 225 rpm for 5 days. Freshly harvested conidia were adjusted to 10^6^ conidia/mL in sterile distilled water before using in experiments. Bird medium, a defined growth medium developed for enhanced conidial germination (49), was used to investigate conditions for biofilm formation.

### Cellular adhesion to a solid surface.

To identify conditions conducive to adherence of cells to a solid hydrophobic surface, we adapted an assay from previous reports for other fungal species (14, 28, 50, 51). Conidia (100 μL of 10^6^ conidia/mL) were inoculated into each well of a 24-well tissue culture plate (Alkali Scientific, Fort Lauderdale, FL) containing 900 μL liquid Bird medium and incubated at RT, shaking at 100 RPM. After 24 h, wells were gently washed three times with sterile distilled water to remove loose cells, and stained for 5 min in 1 mL of crystal violet solution (Sigma Chemical CO, St. Louis, MO; 10 mg/mL), followed by three additional washes in distilled water. For quantification, the cells were destained for 5 min with 1 mL 30% acetic acid, and 10 μL of the supernatant was transferred to a microtiter plate to measure the amount of stain released from the adhered cells. The supernatant was diluted 10-fold with 30% acetic acid to facilitate accurate measuring, and absorbance (590 nm) was measured on a Molecular Devices SpectraMax M2 plate reader (San Jose, CA). For each plate, absorbance was measured only in the center wells to avoid edge effects. Pairwise t-tests were used to assess significance between samples.

### Biofilm development.

To characterize biofilm formation over time, 22 × 22-mm polystyrene coverslips (VWR, Radnor, PA) were cut into quarters, and each quarter was placed in the bottom of a well in a 24-well tissue culture plate containing 900 μL Bird medium, inoculated and cultured as described above to document the full biofilm cycle. To determine the composition of the EPS matrix, macromolecule-specific stains were used to identify and localize components; final concentrations indicated: 10 mg/mL calcofluor white (Fluorescent Brightener 28; Sigma-Aldrich, St. Louis, MO), a nonspecific fluorochrome that stains chitin and cellulose; 10 mg/mL propidium iodide (Sigma-Aldrich, St. Louis, MO), which stains extracellular DNA and RNA; 500 μg/mL nile red (Sigma-Aldrich, St. Louis, MO), which stains lipids; and 2 mg/mL Rhodamine B (Sigma-Aldrich, St. Louis, MO) for staining proteins. Every 4 h following inoculation, eight randomly selected coverslips were removed from the wells, rinsed to clear loose cells, and imaged. To characterize the matrix, three colonized coverslips were selected, and 5 μL of each stain was applied to one of the coverslips, which were then incubated at room temperature in the dark for 5 min. Stained coverslips were rinsed once with distilled water and placed on microscope slides for examination under the microscope, then the experiment was repeated. Images were taken on a Leica DM LB microscope (Leica Inc, Buffalo Grove, IL) with a Zeiss AxioCam HRc camera (Zeiss Inc, Oberkochen, Germany).

Biofilms often develop at air/liquid interfaces. We observed cultures at two different air/liquid interfaces: the surface of Bird medium agar, and the surface of liquid Bird medium. A droplet (10 μL) of WT conidial suspension (10^6^ conidia/mL) was placed in the center of a Petri dish containing Bird medium agar. Development was documented for four consecutive days using a Nikon SMZ800N dissecting microscope and with a Nikon DS-Ri2 camera (Nikon, Tokyo, Japan) at ×20 magnification. From the surface of liquid Bird medium in 24-well plates, floating masses (pellicles) of WT hyphae were harvested, and placed on a slide to observe and photograph at ×10 and ×40 magnification on a Nikon SMZ800N microscope.

### Development of bulbous hyphae.

The WT strain was grown on Bird medium agar for 7 days. To determine conditions for the development of bulbous hyphae, the pH of the medium (normally 6) was adjusted to pH 3 and pH 5 with hydrochloric acid (0.1 M) and to pH 7 and pH 9 with sodium nitrate (0.1 M). To determine if the fungal phenotype was induced by the absence of electron acceptors, Bird medium agar was supplemented with potassium nitrate (Sigma-Aldrich, St. Louis, MO) or Prussian blue (Sigma-Aldrich, St. Louis, MO) at 20 mM, two compounds that act as electron acceptors and have been used previously for this purpose (52, 53). Every 24 h, cells were gently scraped from old growth at the center of the culture, suspended in sterile distilled water, and viewed on a Leica DM LB microscope (Fig. 4). To determine the impact of ROS on biofilm adhesion and formation, hydrogen peroxide (H_2_O_2_, 3% vol/vol; Target, Minneapolis, MN) was added to randomized wells of cultures in liquid Bird medium at 0.1% vol/vol to induce oxidative stress during adhesion assays, as described above.
